# Transcriptomics technologies

**DOI:** 10.1371/journal.pcbi.1005457

**Published:** 2017-05-18

**Authors:** Rohan Lowe, Neil Shirley, Mark Bleackley, Stephen Dolan, Thomas Shafee

**Affiliations:** 1 La Trobe Institute for Molecular Science, La Trobe University, Melbourne, Australia; 2 ARC Centre of Excellence in Plant Cell Walls, University of Adelaide, Adelaide, Australia; 3 Department of Biochemistry, University of Cambridge, Cambridge, United Kingdom

## Abstract

Transcriptomics technologies are the techniques used to study an organism’s transcriptome, the sum of all of its RNA transcripts. The information content of an organism is recorded in the DNA of its genome and expressed through transcription. Here, mRNA serves as a transient intermediary molecule in the information network, whilst noncoding RNAs perform additional diverse functions. A transcriptome captures a snapshot in time of the total transcripts present in a cell.

The first attempts to study the whole transcriptome began in the early 1990s, and technological advances since the late 1990s have made transcriptomics a widespread discipline. Transcriptomics has been defined by repeated technological innovations that transform the field. There are two key contemporary techniques in the field: microarrays, which quantify a set of predetermined sequences, and RNA sequencing (RNA-Seq), which uses high-throughput sequencing to capture all sequences.

Measuring the expression of an organism’s genes in different tissues, conditions, or time points gives information on how genes are regulated and reveals details of an organism’s biology. It can also help to infer the functions of previously unannotated genes. Transcriptomic analysis has enabled the study of how gene expression changes in different organisms and has been instrumental in the understanding of human disease. An analysis of gene expression in its entirety allows detection of broad coordinated trends which cannot be discerned by more targeted assays.

This is a "Topic Page" article for *PLOS Computational Biology*.

## History

**Fig 1:**
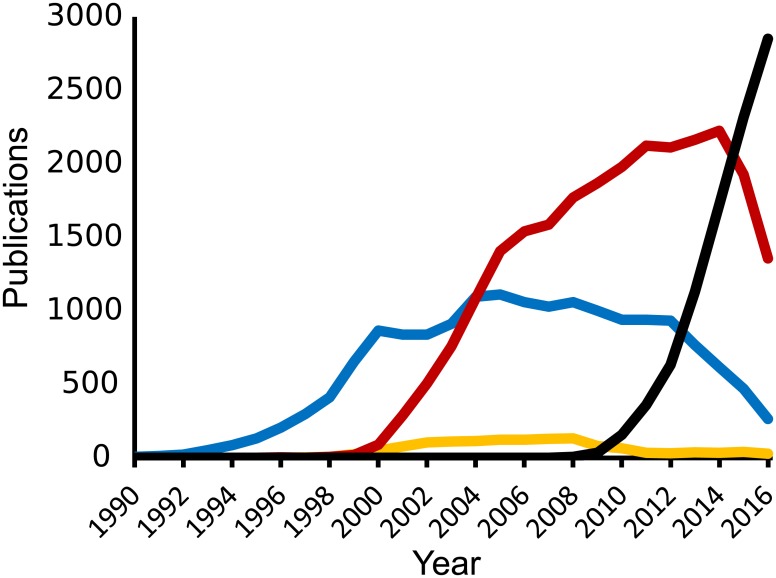
Transcriptomics method use over time. Published papers since 1990, referring to RNA sequencing (black), RNA microarray (red), expressed sequence tag (blue), and serial/cap analysis of gene expression (yellow)[12].

Transcriptomics has been characterised by the development of new techniques which have redefined what is possible every decade or so and render previous technologies obsolete (Fig 1). The first attempt at capturing a partial human transcriptome was published in 1991 and reported 609 mRNA sequences from the human brain [1]. In 2008, two human transcriptomes composed of millions of transcript-derived sequences covering 16,000 genes were published [2][3], and, by 2015, transcriptomes had been published for hundreds of individuals [4][5]. Transcriptomes of different disease states, tissues, or even single cells are now routinely generated [5][6][7]. This explosion in transcriptomics has been driven by the rapid development of new technologies with an improved sensitivity and economy (Table 1) [8][9][10][11].

**Table 1:** Comparison of contemporary methods [23] [24] [10].

Method	RNA-Seq	Microarray
Throughput	High [10]	Higher [10]
Input RNA amount	Low ~ 1 ng total RNA [25]	High ~ 1 μg mRNA [26]
Labour intensity	High (sample preparation and data analysis) [10][23]	Low [10][23]
Prior knowledge	None required, though genome sequence useful [23]	Reference transcripts required for probes [23]
Quantitation accuracy	~90% (limited by sequence coverage) [27]	>90% (limited by fluorescence detection accuracy) [27]
Sequence resolution	Can detect SNPs and splice variants (limited by sequencing accuracy of ~99%) [27]	Dedicated arrays can detect splice variants (limited by probe design and cross-hybridisation) [27]
Sensitivity	10^−6^ (limited by sequence coverage) [27]	10^−3^ (limited by fluorescence detection) [27]
Dynamic range	>10^5^ (limited by sequence coverage) [28]	10^3^−10^4^ (limited by fluorescence saturation) [28]
Technical reproducibility	>99% [29][30]	>99% [31][32]

RNA-Seq, RNA Sequencing

### Before transcriptomics

Studies of individual transcripts were being performed several decades before any transcriptomics approaches were available. Libraries of silkmoth mRNAs were collected and converted to complementary DNA (cDNA) for storage using reverse transcriptase in the late 1970s [13]. In the 1980s, low-throughput Sanger sequencing began to be used to sequence random individual transcripts from these libraries, called expressed sequence tags (ESTs) [2][14][15][16]. The Sanger method of sequencing was predominant until the advent of high-throughput methods such as sequencing by synthesis (Solexa/Illumina, San Diego, CA). ESTs came to prominence during the 1990s as an efficient method to determine the gene content of an organism without sequencing the entire genome [16]. Quantification of individual transcripts by northern blotting, nylon membrane arrays, and later reverse transcriptase quantitative PCR (RT-qPCR) were also popular [17][18], but these methods are laborious and can only capture a tiny subsection of a transcriptome [12]. Consequently, the manner in which a transcriptome as a whole is expressed and regulated remained unknown until higher-throughput techniques were developed.

### Early attempts

The word “transcriptome” was first used in the 1990s [19][20]. In 1995, one of the earliest sequencing-based transcriptomic methods was developed, serial analysis of gene expression (SAGE), which worked by Sanger sequencing of concatenated random transcript fragments [21]. Transcripts were quantified by matching the fragments to known genes. A variant of SAGE using high-throughput sequencing techniques, called digital gene expression analysis, was also briefly used [9][22]. However, these methods were largely overtaken by high throughput sequencing of entire transcripts, which provided additional information on transcript structure, e.g., splice variants [9].

### Development of contemporary techniques

The dominant contemporary techniques, microarrays and RNA-Seq, were developed in the mid-1990s and 2000s [9][33]. Microarrays that measure the abundances of a defined set of transcripts via their hybridisation to an array of complementary probes were first published in 1995 [34][35]. Microarray technology allowed the assay of thousands of transcripts simultaneously at a greatly reduced cost per gene and labour saving [36]. Both spotted oligonucleotide arrays and Affymetrix (Santa Clara, California) high-density arrays were the method of choice for transcriptional profiling until the late 2000s [12][33]. Over this period, a range of microarrays were produced to cover known genes in model or economically important organisms. Advances in design and manufacture of arrays improved the specificity of probes and allowed for more genes to be tested on a single array. Advances in fluorescence detection increased the sensitivity and measurement accuracy for low abundance transcripts [35][37].

RNA-Seq refers to the sequencing of transcript cDNAs, in which abundance is derived from the number of counts from each transcript. The technique has therefore been heavily influenced by the development of high-throughput sequencing technologies [9][11]. Massively parallel signature sequencing (MPSS) was an early example based on generating 16–20 bp sequences via a complex series of hybridisations [38] and was used in 2004 to validate the expression of 10^4^ genes in *Arabidopsis thaliana* [39]. The earliest RNA-Seq work was published in 2006 with 10^5^ transcripts sequenced using the 454 technology [40]. This was sufficient coverage to quantify relative transcript abundance. RNA-Seq began to increase in popularity after 2008 when new Solexa/Illumina technologies (San Diego, CA) allowed 10^9^ transcript sequences to be recorded [4][10][41][42]. This yield is now sufficient for accurate quantitation of entire human transcriptomes.

## Data gathering

Generating data on RNA transcripts can be achieved via either of two main principles: sequencing of individual transcripts (ESTs, or RNA-Seq), or hybridisation of transcripts to an ordered array of nucleotide probes (i.e., microarrays).

### Isolation of RNA

All transcriptomic methods require RNA to first be isolated from the experimental organism before transcripts can be recorded. Although biological systems are incredibly diverse, RNA extraction techniques are broadly similar and involve the following: mechanical disruption of cells or tissues, disruption of RNase with chaotropic salts [43], disruption of macromolecules and nucleotide complexes, separation of RNA from undesired biomolecules including DNA, and concentration of the RNA via precipitation from solution or elution from a solid matrix [43][44]. Isolated RNA may additionally be treated with DNase to digest any traces of DNA [45]. It is necessary to enrich messenger RNA as total RNA extracts are typically 98% ribosomal RNA [46]. Enrichment for transcripts can be performed by poly-A affinity methods or by depletion of ribosomal RNA using sequence-specific probes [47]. Degraded RNA may affect downstream results; for example, mRNA enrichment from degraded samples will result in the depletion of 5ʹ mRNA ends and uneven signal across the length of a transcript. Snap-freezing of tissue prior to RNA isolation is typical, and care is taken to reduce exposure to RNase enzymes once isolation is complete [44].

### EST

An EST is a short nucleotide sequence generated from a single RNA transcript. RNA is first copied as cDNA by a reverse transcriptase enzyme before the resultant cDNA is sequenced [16]. The Sanger method of sequencing was predominant until the advent of high-throughput methods such as sequencing by synthesis (Solexa/Illumina, San Diego, CA). Because ESTs don't require prior knowledge of the organism from which they come, they can also be made from mixtures of organisms or environmental samples [16]. Although higher-throughput methods are now used, EST libraries commonly provided sequence information for early microarray designs; for example, a barley GeneChip was designed from 350,000 previously sequenced ESTs [48].

### Serial and Cap analysis of gene expression (SAGE/CAGE)

**Fig 2:**
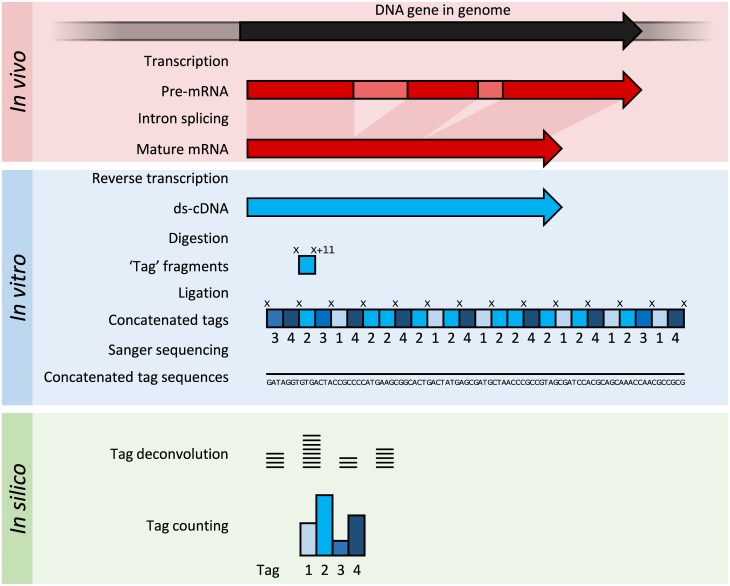
Summary of SAGE. Within the organisms, genes are transcribed and spliced (in eukaryotes) to produce mature mRNA transcripts (red). The mRNA is extracted from the organism, and reverse transcriptase is used to copy the mRNA into stable double-stranded–cDNA (ds-cDNA; blue). In SAGE, the ds-cDNA is digested by restriction enzymes (at location “X” and “X”+11) to produce 11-nucleotide “tag” fragments. These tags are concatenated and sequenced using long-read Sanger sequencing (different shades of blue indicate tags from different genes). The sequences are deconvoluted to find the frequency of each tag. The tag frequency can be used to report on transcription of the gene that the tag came from.

SAGE was a development of EST methodology to increase the throughput of the tags generated and allow some quantitation of transcript abundance (Fig 2) [21]. cDNA is generated from the RNA but is then digested into 11 bp “tag” fragments using restriction enzymes that cut at a specific sequence, and 11 base pairs along from that sequence. These cDNA tags are then concatenated head-to-tail into long strands (>500 bp) and sequenced using low-throughput, but long read length methods such as Sanger sequencing. Once the sequences are deconvoluted into their original 11 bp tags [21]. If a reference genome is available, these tags can sometimes be aligned to identify their corresponding gene. If a reference genome is unavailable, the tags can simply be directly used as diagnostic markers if found to be differentially expressed in a disease state.

The Cap analysis of gene expression (CAGE) method is a variant of SAGE that sequences tags from the 5ʹ end of an mRNA transcript only [49]. Therefore, the transcriptional start site of genes can be identified when the tags are aligned to a reference genome. Identifying gene start sites is of use for promoter analysis and for the cloning of full-length cDNAs.

SAGE and CAGE methods produce information on more genes than was possible when sequencing single ESTs, but the sample preparation and data analysis are typically more labour intensive.

### Microarrays

#### Principles and advances

**Fig 3:**
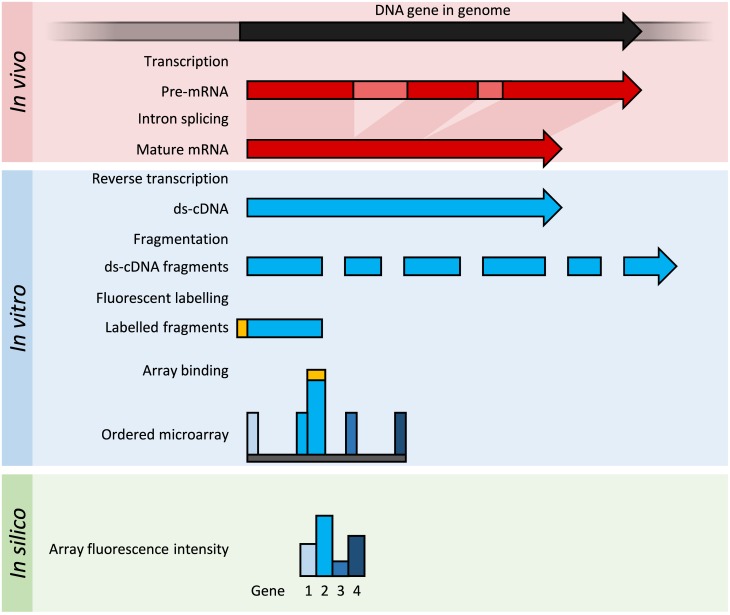
Summary of DNA microarrays. Within the organisms, genes are transcribed and spliced (in eukaryotes) to produce mature mRNA transcripts (red). The mRNA is extracted from the organism and reverse transcriptase is used to copy the mRNA into stable double-stranded–cDNA (ds-cDNA; blue). In microarrays, the ds-cDNA is fragmented and fluorescently labelled (orange). The labelled fragments bind to an ordered array of complementary oligonucleotides, and measurement of fluorescent intensity across the array indicates the abundance of a predetermined set of sequences. These sequences are typically specifically chosen to report on genes of interest within the organism's genome.

Microarrays consist of short nucleotide oligomers, known as "probes," which are arrayed on a solid substrate (e.g., glass) [50]. Transcript abundance is determined by hybridisation of fluorescently labelled transcripts to these probes (Fig 3) [51]. The fluorescence intensity at each probe location on the array indicates the transcript abundance for that probe sequence [51]. Microarrays require some prior knowledge of the organism of interest, for example, in the form of an annotated genome sequence or in a library of ESTs that can be used to generate the probes for the array.

#### Methods

The manufacture of microarrays relies on micro and nanofabrication techniques. Microarrays for transcriptomics typically fall into one of the following two broad categories: low-density spotted arrays or high-density short probe arrays [36]. Transcript presence may be recorded with single- or dual-channel detection of fluorescent tags.

Spotted low-density arrays typically feature picolitre drops of a range of purified cDNAs arrayed on the surface of a glass slide [52]. The probes are longer than those of high-density arrays and typically lack the transcript resolution of high-density arrays. Spotted arrays use different fluorophores for test and control samples, and the ratio of fluorescence is used to calculate a relative measure of abundance [53]. High-density arrays use single channel detection, and each sample is hybridised and detected individually [54]. High-density arrays were popularised by the Affymetrix GeneChip array (Santa Clara, CA), in which each transcript is quantified by several short 25-mer probes that together assay one gene [55].

NimbleGen arrays (Pleasanton, CA) are high-density arrays produced by a maskless-photochemistry method, which permits flexible manufacture of arrays in small or large numbers. These arrays have hundreds of thousands of 45- to 85-mer probes and are hybridised with a one-colour labelled sample for expression analysis [56]. Some designs incorporate up to 12 independent arrays per slide.

### RNA-Seq

#### Principles and advances

**Fig 4:**
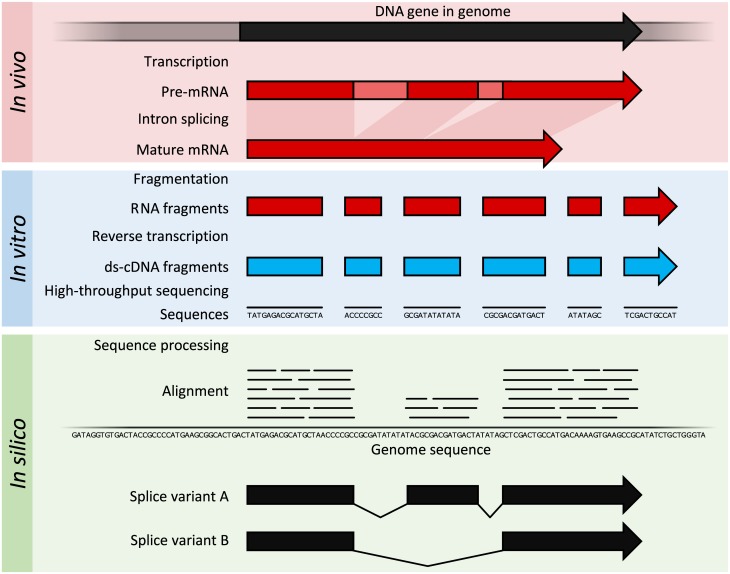
Summary of RNA sequencing. Within the organisms, genes are transcribed and spliced (in eukaryotes) to produce mature mRNA transcripts (red). The mRNA is extracted from the organism, fragmented and copied into stable double-stranded–cDNA (ds-cDNA; blue). The ds-cDNA is sequenced using high-throughput, short-read sequencing methods. These sequences can then be aligned to a reference genome sequence to reconstruct which genome regions were being transcribed. These data can be used to annotate where expressed genes are, their relative expression levels, and any alternative splice variants.

RNA-Seq refers to the combination of a high-throughput sequencing methodology with computational methods to capture and quantify transcripts present in an RNA extract (Fig 4) [10]. The nucleotide sequences generated are typically around 100 bp in length, but can range from 30 bp to over 10,000 bp, depending on the sequencing method used. RNA-Seq leverages deep sampling of the transcriptome with many short fragments from a transcriptome to allow computational reconstruction of the original RNA transcript by aligning reads to a reference genome or to each other (de novo assembly) [9]. The typical dynamic range of 5 orders of magnitude for RNA-Seq is a key advantage over microarray transcriptomes. In addition, input RNA amounts are much lower for RNA-Seq (nanogram quantity) compared to microarrays (microgram quantity), which allowed finer examination of cellular structures, down to the single-cell level when combined with linear amplification of cDNA [25]. Theoretically, there is no upper limit of quantification in RNA-Seq, and background signal is very low for 100 bp reads in nonrepetitive regions [10].

RNA-Seq may be used to identify genes within a genome or identify which genes are active at a particular point in time, and read counts can be used to accurately model the relative gene expression level. RNA-Seq methodology has constantly improved, primarily through the development of DNA sequencing technologies to increase throughput, accuracy, and read length [57]. Since the first descriptions in 2006 and 2008 [40][58], RNA-Seq has been rapidly adopted and overtook microarrays as the dominant transcriptomics technique in 2015 [59].

The quest for transcriptome data at the level of individual cells has driven advances in RNA-Seq library preparation methods, resulting in dramatic advances in sensitivity. Single-cell transcriptomes are now well described and have even been extended to in situ RNA-Seq where transcriptomes of individual cells are directly interrogated in fixed tissues [60].

#### Methods

RNA-Seq was established in concert with the rapid development of a range of high-throughput DNA sequencing technologies [61]. However, before the extracted RNA transcripts are sequenced, several key processing steps are performed. Methods differ in the use of transcript enrichment, fragmentation, amplification, single or paired-end sequencing, and whether to preserve strand information.

The sensitivity of an RNA-Seq experiment can be increased by enriching classes of RNA that are of interest and depleting known abundant RNAs. The mRNA molecules can be separated by using oligonucleotides probes which bind their poly-A tails. Alternatively, ribo-depletion can be used to specifically remove abundant but uninformative ribosomal RNAs (rRNAs) by hybridisation to probes tailored to the taxon's specific rRNA sequences (e.g., mammal rRNA, plant rRNA). However, ribo-depletion can also introduce some bias via nonspecific depletion of off-target transcripts [62]. Small RNAs such as microRNAs, can be purified based on their size by gel electrophoresis and extraction.

Because mRNAs are longer than the read-lengths of typical high-throughput sequencing methods, transcripts are usually fragmented prior to sequencing. The fragmentation method is a key aspect of sequencing library construction [63]. It may incorporate chemical hydrolysis, nebulisation, or sonication of RNA, or utilise simultaneous fragmentation, and tagging of cDNA by transposase enzymes.

During preparation for sequencing, cDNA copies of transcripts may be amplified by PCR to enrich for fragments that contain the expected 5ʹ and 3ʹ adapter sequences [64]. Amplification is also used to allow sequencing of very low-input amounts of RNA, down to as little as 50 pg in extreme applications [65]. Spike-in controls can be used to provide quality control assessment of library preparation and sequencing, in terms of guanine-cytosine content, fragment length, as well as the bias due to fragment position within a transcript [66]. Unique molecular identifiers (UMIs) are short random sequences that are used to individually tag sequence fragments during library preparation so that every tagged fragment is unique [67]. UMIs provide an absolute scale for quantification and the opportunity to correct for subsequent amplification bias introduced during library construction and accurately estimate the initial sample size. UMIs are particularly well-suited to single-cell RNA-Seq transcriptomics, in which the amount of input RNA is restricted and extended amplification of the sample is required [68][69][70].

Once the transcript molecules have been prepared, they can be sequenced in just one direction (single-end) or both directions (paired-end). A single-end sequence is usually quicker to produce, cheaper than paired-end sequencing, and sufficient for quantification of gene expression levels. Paired-end sequencing produces more robust alignments and/or assemblies, which is beneficial for gene annotation and transcript isoform discovery [10]. Strand-specific RNA-Seq methods preserve the strand information of a sequenced transcript [71]. Without strand information, reads can be aligned to a gene locus, but do not inform in which direction the gene is transcribed. Stranded-RNA-Seq is useful for deciphering transcription for genes that overlap in different directions, and to make more robust gene predictions in nonmodel organisms [71].

Currently, RNA-Seq relies on copying of RNA molecules into cDNA molecules prior to sequencing; hence, the subsequent platforms are the same for transcriptomic and genomic data (Table 2). Consequently, the development of DNA sequencing technologies has been a defining feature of RNA-Seq [73][75][76]. Direct sequencing of RNA using nanopore sequencing represents a current state-of-the-art RNA-Seq technique in its infancy (in pre-release beta testing as of 2016) [77][78]. However, nanopore sequencing of RNA can detect modified bases that would be otherwise masked when sequencing cDNA and also eliminates amplification steps that can otherwise introduce bias [11][79].

**Table 2:** Sequencing technology platforms commonly used for RNA-Seq [72][73].

Platform (Manufacturer)	Commercial release	Typical read length	Maximum throughput per run	Single read accuracy	RNA-Seq runs deposited in the NCBI SRA (Oct 2016) [74]
454 (Roche, Basel, Switzerland)	2005	700 bp	0.7 Gbp	99.9%	3548
Illumina (‎Illumina, San Diego, CA, USA)	2006	50–300 bp	900 Gbp	99.9%	362903
SOLiD (Thermo Fisher Scientific, Waltham, MA, USA)	2008	50 bp	320 Gbp	99.9%	7032
Ion Torrent (Thermo Fisher Scientific, Waltham, MA, USA)	2010	400 bp	30 Gbp	98%	1953
PacBio (Pacbio, Menlo Park, CA, USA)	2011	10,000 bp	2 Gbp	87%	160

NCBI, National Center for Biotechnology Information; SRA, Sequence Read Archive; RNA-Seq, RNA sequencing.

The sensitivity and accuracy of an RNA-Seq experiment are dependent on the number of reads obtained from each sample. A large number of reads are needed to ensure sufficient coverage of the transcriptome, enabling detection of low abundance transcripts. Experimental design is further complicated by sequencing technologies with a limited output range, the variable efficiency of sequence creation, and variable sequence quality. Added to those considerations is that every species has a different number of genes and therefore requires a tailored sequence yield for an effective transcriptome. Early studies determined suitable thresholds empirically, but as the technology matured, suitable coverage is predicted computationally by transcriptome saturation. Somewhat counterintuitively, the most effective way to improve detection of differential expression in low expression genes is to add more biological replicates, rather than adding more reads [80]. The current benchmarks recommended by the Encyclopedia of DNA Elements (ENCODE) Project are for 70-fold exome coverage for standard RNA-Seq and up to 500-fold exome coverage to detect rare transcripts and isoforms [81][82][83].

## Data analysis

Transcriptomics methods are highly parallel and require significant computation to produce meaningful data for both microarray and RNA-Seq experiments. Microarray data are recorded as high-resolution images, requiring feature detection and spectral analysis. Microarray raw image files are each about 750 MB in size, while the processed intensities are around 60 MB in size. Multiple short probes matching a single transcript can reveal details about the intron-exon structure, requiring statistical models to determine the authenticity of the resulting signal. RNA-Seq studies can produce >10^9^ of short DNA sequences, which must be aligned to reference genomes comprised of millions to billions of base pairs. De novo assembly of reads within a dataset requires the construction of highly complex sequence graphs. RNA-Seq operations are highly repetitious and benefit from parallelised computation, but modern algorithms mean consumer computing hardware is sufficient for simple transcriptomics experiments that do not require de novo assembly of reads. A human transcriptome could be accurately captured by using RNA-Seq with 30 million 100 bp sequences per sample [84][85]. This example would require approximately 1.8 gigabytes of disk space per sample when stored in a compressed fastq format. Processed count data for each gene would be much smaller, equivalent to processed microarray intensities. Sequence data may be stored in public repositories, such as the Sequence Read Archive (SRA) [86]. RNA-Seq datasets can be uploaded via the Gene Expression Omnibus.

### Image processing

**Fig 5:**
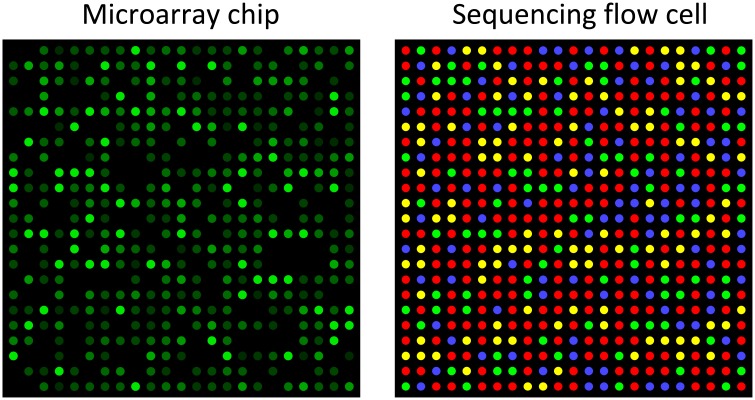
Microarray and sequencing flow cell. Microarrays and RNA sequencing (RNA-Seq) rely on image analysis in different ways. In a microarray chip, each spot on a chip is a defined oligonucleotide probe, and fluorescence intensity directly detects the abundance of a specific sequence (Affymetrix, Santa Clara, CA). In a high-throughput sequencing flow cell, spots are sequenced one nucleotide at a time, with the colour at each round indicating the next nucleotide in the sequence (Illumina Hiseq, San Diego, CA). Other variations of these techniques use more or fewer colour channels.

Microarray image processing must correctly identify the regular grid of features within an image and independently quantify the fluorescence intensity for each feature (Fig 5). Image artefacts must be additionally identified and removed from the overall analysis [87]. Fluorescence intensities directly indicate the abundance of each sequence because the sequence of each probe on the array is already known.

The first steps of RNA-seq also include similar image processing, however conversion of images to sequence data is typically handled automatically by the instrument software. The Illumina sequencing-by-synthesis method results in a random or ordered array of clusters distributed over the surface of a flow cell. The flow cell is imaged up to four times during each sequencing cycle, with tens to hundreds of cycles in total. Flow cell clusters are analogous to microarray spots and must be correctly identified during the early stages of the sequencing process. In Roche‘s Pyrosequencing method, the intensity of emitted light determines the number of consecutive nucleotides in a homopolymer repeat. There are many variants on these methods, each with a different error profile for the resulting data [88].

### RNA-Seq data analysis

RNA-Seq experiments generate a large volume of raw sequence reads, which have to be processed to yield useful information. Data analysis usually requires a combination of bioinformatics software tools that vary according to the experimental design and goals. The process can be broken down into the following four stages: quality control, alignment, quantification, and differential expression [89]. Most popular RNA-Seq programs are run from a command-line interface, either in a Unix environment or within the R/Bioconductor statistical environment [90].

#### Quality control

Sequence reads are not perfect, so the accuracy of each base in the sequence needs to be estimated for downstream analyses. Raw data are examined for high quality scores for base calls, guanine-cytosine content matches the expected distribution, the over representation of particularly short sequence motifs (k-mers), and an unexpectedly high read duplication rate [85]. Several options exist for sequence quality analysis, including the FastQC and FaQCs software packages [91][92]. Abnormalities identified may be removed by trimming or tagged for special treatment during later processes.

#### Alignment

In order to link sequence read abundance to expression of a particular gene, transcript sequences are aligned to a reference genome, or de novo aligned to one another if no reference is available. The key challenges for alignment software include sufficient speed to permit >10^9^ of short sequences to be aligned in a meaningful timeframe, flexibility to recognise and deal with intron splicing of eukaryotic mRNA, and correct assignment of reads that map to multiple locations. Software advances have greatly addressed these issues, and increases in sequencing read length are further reducing multimapping reads. A list of currently available high-throughput sequence aligners is maintained by the EBI [93][94].

Alignment of primary transcript mRNA sequences derived from eukaryotes to a reference genome requires specialised handling of intron sequences, which are absent from mature mRNA. Short read aligners perform an additional round of alignments specifically designed to identify splice junctions, informed by canonical splice site sequences and known intron splice site information. Identification of intron splice junctions prevents reads from being misaligned across splice junctions or erroneously discarded, allowing for more reads to be aligned to the reference genome and improving the accuracy of gene expression estimates. Because gene regulation may occur at the mRNA isoform level, splice-aware alignments also permit detection of isoform abundance changes that would otherwise be lost in a bulked analysis [95].

De novo assembly can be used to align reads to one another to construct full-length transcript sequences without the use of a reference genome (Table 3) [96]. Challenges particular to de novo assembly include larger computational requirements compared to a reference-based transcriptome, additional validation of gene variants or fragments, additional annotation of assembled transcripts. The first metrics used to describe transcriptome assemblies, such as N50, have been shown to be misleading [97], and subsequently improved evaluation methods are now available [98][99]. Annotation-based metrics are better assessments of assembly completeness, such as contig reciprocal best hit count. Once assembled de novo, the assembly can be used as a reference for subsequent sequence alignment methods and quantitative gene expression analysis.

**Table 3:** RNA-Seq de novo assembly software.

Software (Manufacturer)	Released	Last Updated	Resource load	Strengths and weaknesses
Velvet-Oases [100][101]	2008	2011	Heavy	The original short read assembler, now largely superseded.
SOAPdenovo-trans [102]	2011	2015	Moderate	Early short read assembler, updated for transcript assembly.
Trans-ABySS [103]	2010	2016	Moderate	Short reads, large genomes, MPI-parallel version available.
Trinity [104][105]	2011	2017	Moderate	Short reads, large genomes, memory intensive.
miraEST [106]	1999	2016	Moderate	Repetitive sequences, hybrid data input, wide range of sequence platforms accepted.
Newbler [107]	2004	2012	Heavy	Specialised for Roche 454 sequence, homo-polymer error handling.
CLC genomics workbench (Qiagen—Venlo, Netherlands) [108]	2008	2014	Light	Graphical user interface, hybrid data.

MPI, Message Passing Interface; RNA-Seq, RNA sequencing.

#### Quantification

Quantification of sequence alignments may be performed at the gene, exon, or transcript level. Typical outputs include a table of reads counts for each feature supplied to the software, for example, for genes in a general feature format file. Gene and exon read counts may be calculated easily using the HTSeq software package, for example [109]. Quantitation at the transcript level is more complicated and requires probabilistic methods to estimate transcript isoform abundance from short read information, for example, using cufflinks software [95]. Reads that align equally well to multiple locations must be identified and either removed, aligned to one of the possible locations, or aligned to the most probable location.

Some quantification methods can circumvent the need for an exact alignment of a read to a reference sequence all together. The kallisto method combines pseudoalignment and quantification into a single step that runs 2 orders of magnitude faster than comparable methods such as tophat/cufflinks, with less computational burden [110].

#### Differential expression

**Fig 6:**
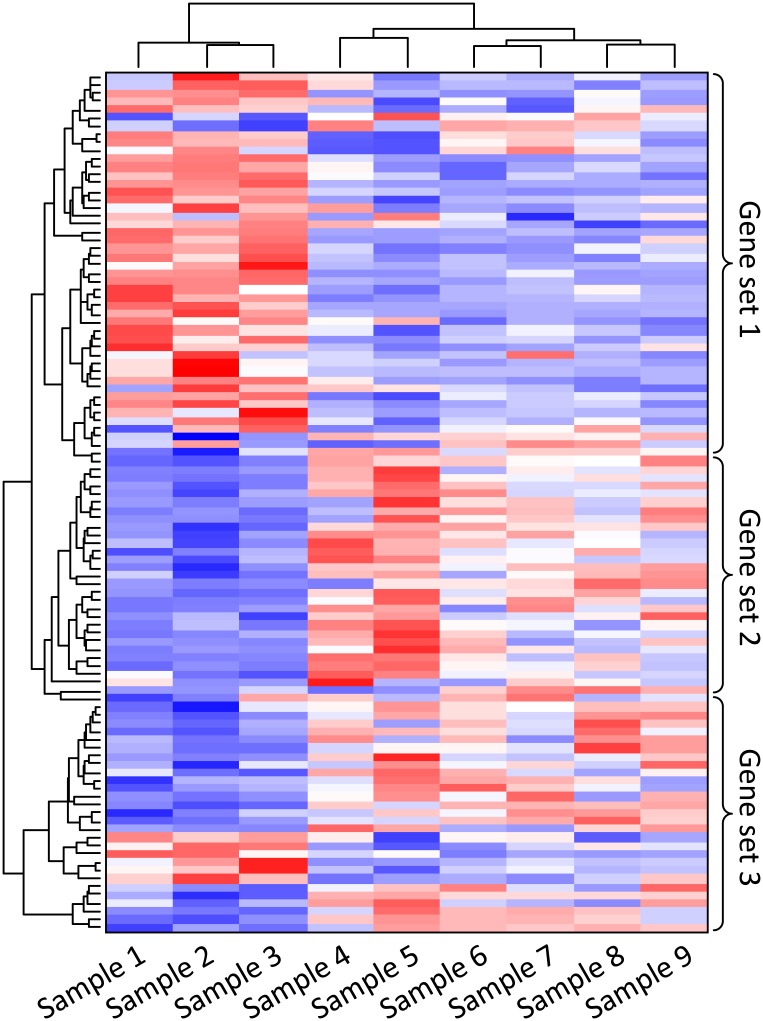
identification of gene co-expression patterns across different samples. Heatmap Each column contains the measurements for gene expression change for a single sample. Relative gene expression is indicated by colour: high-expression (red), median-expression (white) and low-expression (blue). Genes and samples with similar expression profiles can be automatically grouped (left and top trees). Samples may be different individuals, tissues, environments, or health conditions. In this example, expression of gene set 1 is high and expression of gene set 2 is low in samples 1, 2, and 3.

Once quantitative counts of each transcript are available, differential gene expression is then measured by normalising, modelling, and statistically analysing the data (Fig 6). Examples of dedicated software are described in Table 4. Most read a table of genes and read counts as their input, but some, such as cuffdiff, will accept binary alignment map format read alignments as input. The final outputs of these analyses are gene lists with associated pair-wise tests for differential expression between treatments and the probability estimates of those differences.

**Table 4:** RNA-Seq differential gene expression software.

Software	Environment	Specialisation
Cuffdiff2 [111]	Unix-based	Transcript analysis at isoform-level
EdgeR [112]	R/Bioconductor	Any count-based genomic data
DEseq2 [113]	R/Bioconductor	Flexible data types, low replication
Limma/Voom [114]	R/Bioconductor	Microarray or RNA-Seq data, isoform analysis, flexible experiment design

RNA-Seq, RNA sequencing.

### Validation

Transcriptomic analyses may be validated using an independent technique, for example, quantitative PCR (qPCR), which is recognisable and statistically assessable [115]. Gene expression is measured against defined standards both for the gene of interest and control genes. The measurement by qPCR is similar to that obtained by RNA-Seq wherein a value can be calculated for the concentration of a target region in a given sample. qPCR is, however, restricted to amplicons smaller than 300 bp, usually toward the 3ʹ end of the coding region, avoiding the 3ʹ unstralated region (3ʹUTR) [116]. If validation of transcript isoforms is required, an inspection of RNA-Seq read alignments should indicate where qPCR primers might be placed for maximum discrimination. The measurement of multiple control genes along with the genes of interest produces a stable reference within a biological context [117]. qPCR validation of RNA-Seq data has generally shown that different RNA-Seq methods are highly correlated [58][118][119].

Functional validation of key genes is an important consideration for post transcriptome planning. Observed gene expression patterns may be functionally linked to a phenotype by an independent knock-down/rescue study in the organism of interest.

## Applications

### Diagnostics and disease profiling

Transcriptomic strategies have seen broad application across diverse areas of biomedical research, including disease diagnosis and profiling [10]. RNA-Seq approaches have allowed for the large-scale identification of transcriptional start sites and uncovered alternative promoter usage and novel splicing alterations. These regulatory elements are important in human disease, and therefore, defining such variants is crucial to the interpretation of disease-association studies [120]. RNA-Seq can also identify disease-associated single nucleotide polymorphisms (SNP), allele-specific expression, and gene fusions, contributing to our understanding of disease causal variants [121].

Retrotransposons are transposable elements which proliferate within eukaryotic genomes through a process involving reverse transcription. RNA-Seq can provide information about the transcription of endogenous retrotransposons that may influence the transcription of neighbouring genes by various epigenetic mechanisms that lead to disease [122]. Similarly, the potential for using RNA-Seq to understand immune-related disease is expanding rapidly due to the ability to dissect immune cell populations and to sequence T cell and B cell receptor repertoires from patients [123][124].

### Human and pathogen transcriptomes

RNA-Seq of human pathogens has become an established method for quantifying gene expression changes, identifying novel virulence factors, predicting antibiotic resistance, and unveiling host-pathogen immune interactions [125][126]. A primary aim of this technology is to develop optimised infection control measures and targeted, individualised treatment [124].

Transcriptomic analysis has predominantly focused on either the host or the pathogen. Dual RNA-Seq has recently been applied to simultaneously profile RNA expression in both the pathogen and host throughout the infection process. This technique enables the study of the dynamic response and interspecies gene regulatory networks in both interaction partners from initial contact through to invasion and the final persistence of the pathogen or clearance by the host immune system [127][128].

### Responses to environment

Transcriptomics allows for the identification of genes and pathways that respond to and counteract biotic and abiotic environmental stresses. The nontargeted nature of transcriptomics allows for the identification of novel transcriptional networks in complex systems. For example, comparative analysis of a range of chickpea lines at different developmental stages identified distinct transcriptional profiles associated with drought and salinity stresses, including identifying the role of transcript isoforms of Apetela 2 and Ethylene-Responsive Element Binding Protein (AP2-EREBP) [129]. Investigation of gene expression during biofilm formation by the fungal pathogen *Candida albican*s revealed a coregulated set of genes critical for biofilm establishment and maintenance [130].

Transcriptomic profiling also provides crucial information on mechanisms of drug resistance. Analysis of over a thousand *Plasmodium falciparum* isolates identified that upregulation of the unfolded protein response and slower progression through the early stages of the asexual intraerythrocytic developmental cycle were associated with artemisinin resistance in isolates from Southeast Asia [131].

### Gene function annotation

All transcriptomic techniques have been particularly useful in identifying the functions of genes and identifying those responsible for particular phenotypes. Transcriptomics of *Arabidopsis* ecotypes that hyperaccumulate metals correlated genes involved in metal uptake, tolerance, and homeostasis with the phenotype [132]. Integration of RNA-Seq datasets across different tissues has been used to improve annotation of gene functions in commercially important organisms (e.g., cucumber) [133] or threatened species (e.g., koala) [134].

Assembly of RNA-Seq reads is not dependent on a reference genome [104], and it is so ideal for gene expression studies of nonmodel organisms with nonexisting or poorly developed genomic resources. For example, a database of SNPs used in Douglas fir breeding programs was created by de novo transcriptome analysis in the absence of a sequenced genome [135]. Similarly, genes that function in the development of cardiac, muscle, and nervous tissue in lobster were identified by comparing the transcriptomes of the various tissue types without use of a genome sequence [136]. RNA-Seq can also be used to identify previously unknown protein coding regions in existing sequenced genomes.

### Noncoding RNA

Transcriptomics is most commonly applied to the mRNA content of the cell. However, the same techniques are equally applicable to noncoding RNAs that are not translated into a protein, but instead, have direct functions (e.g., roles in protein translation, DNA replication, RNA splicing, and transcriptional regulation) [137][138][139][140]. Many of these noncoding RNAs affect disease states, including cancer, cardiovascular, and neurological diseases [141].

## Transcriptome databases

Transcriptomics studies generate large amounts of data that has potential applications far beyond the original aims of an experiment. As such, raw or processed data may be deposited into public databases to ensure their utility for the broader scientific community (Table 5). For example, as of 2016, the Gene Expression Omnibus contained millions of experiments.

**Table 5:** Transcriptomic databases.

Name	Host	Data	Description
Gene Expression Omnibus [142]	NCBI	Microarray RNA-Seq	First transcriptomics database to accept data from any source. Introduced MIAME and MINSEQE community standards that define necessary experiment metadata to ensure effective interpretation and repeatability [143][144].
ArrayExpress [145]	ENA	Microarray	Imports datasets from the Gene Expression Omnibus and accepts direct submissions. Processed data and experiment metadata are stored at ArrayExpress, while the raw sequence reads are held at the ENA. Complies with MIAME and MINSEQE standards [144][145].
Expression Atlas [146]	EBI	Microarray RNA-Seq	Tissue-specific gene expression database for animals and plants. Displays secondary analyses and visualisation, such as functional enrichment of Gene Ontology terms, InterPro domains, or pathways. Links to protein abundance data where available.
Genevestigator [147]	Privately curated	Microarray RNA-Seq	Contains manual curations of public transcriptome datasets, focusing on medical and plant biology data. Individual experiments are normalised across the full database, to allow comparison of gene expression across diverse experiments. Full functionality requires licence purchase, with free access to a limited functionality.
RefEx [148]	DDBJ	All	Human, mouse, and rat transcriptomes from 40 different organs. Gene expression visualised as heatmaps projected onto 3D representations of anatomical structures.
NONCODE [149]	noncode.org	RNA-Seq	ncRNAs excluding tRNA and rRNA.

DDBJ, DNA Data Bank of Japan; EBI, European Bioinformatics Institute; ENA, European Nucleotide Archive; MIAME, Minimum Information About a Microarray Experiment; MINSEQE, Minimum Information about a high-throughput nucleotide SEQuencing Experiment; NCBI, National Center for Biotechnology Information; ncRNAs, noncoding RNAs; RNA-Seq, RNA sequencing.

## Conclusions

Transcriptomics has revolutionised our understanding of how genomes are expressed. Over the last three decades, new technologies have redefined what is possible to investigate, and integration with other omics technologies is giving an increasingly integrated view of the complexities of cellular life. The plummeting cost of transcriptomics studies have made them possible for small laboratories, and large-scale transcriptomics consortia are able to undertake experiments comparing transcriptomes of thousands of organisms, tissues, or environmental conditions. This trend is likely to continue as sequencing technologies improve.

## Supporting information

S1 Text Version history of the text file. (XML)

S2 Text Peer reviews and response to reviews. (XML)
